# User-Centered Development of a Web Platform Supporting Community-Based Health Care Organizations for Older Persons in Need of Support: Qualitative Focus Group Study

**DOI:** 10.2196/24006

**Published:** 2021-03-10

**Authors:** Verena Biehl, Heidrun Becker, Alenka Ogrin, Alenka Reissner, Johannes Burger, Andrea Glaessel

**Affiliations:** 1 Institute of Health Sciences Zurich University of Applied Sciences Winterthur Switzerland; 2 Research and Development in Occupational Therapy Zurich University of Applied Sciences Winterthur Switzerland; 3 Zveza društev upokojencev Slovenije (ZDUS) Ljubljana Slovenia; 4 SYNYO GmbH Vienna Austria; 5 Institute of Biomedical Ethics and History of Medicine University of Zurich Zurich Switzerland

**Keywords:** community-based health care services, older persons in need of support, user-centered design, focus groups, qualitative research, web platform

## Abstract

**Background:**

The ongoing changes in population demographics increase the relevance of dignified aging across Europe. Community-based health care (CBHC) organizations are necessary to provide sustainable strategies for organizing care for older persons in need of support. To support the digitalization of these organizations, new business models and suitable web platforms are necessary.

**Objective:**

This study, which is part of the European Active and Assisted Living (AAL) project called “ICareCoops”, aimed to explore concepts, approaches, and workflows of CBHC organizations to achieve a comprehensive understanding of extant services offered and relevant requirements to support these services with information and computer technology (ICT) solutions.

**Methods:**

A qualitative study with six focus groups (FGs) with 40 participants was conducted in Switzerland and Slovenia to identify potential stakeholders’ needs and requirements for the user-centered development of a web platform. Data were collected from three different stakeholder groups: (1) older persons in need of support as care receivers, (2) significant others of older persons in need of support, and (3) managers or care providers of CBHC organizations. A semistructured interview guide with open questions was used for data collection. FG sessions were audio-recorded and transcribed verbatim. Thematic content analysis was used to analyze the content of the FG sessions. To assist with further web platform development, the responses of the FG participants were translated into user stories to describe technical requirements.

**Results:**

By analyzing the transcripts, five main categories were identified: (1) ICT usage behavior of users, (2) challenges of web platform usage, (3) content and technical requirements for the web platform, (4) form and services of CBHC organizations, and (5) rationales of CBHC organizations. The main issues identified were the need for seniors to have individual contact with the CBHC organization and the possibility to coordinate routine services via the web platform, such as ordering meals-on-wheels or booking a caregiver to accompany an older person to the doctor.

**Conclusions:**

The majority of participants showed a lack of familiarity with the usage of ICT. Nevertheless, they were open-minded regarding web platform usage to facilitate workflows and to benefit CBHC organizations. Cooperatives as an organizational model demonstrate a high potential to address users’ needs. Therefore, the web platform offers an essential tool for innovative health care models in the future. Searching for care services, contacting care providers, and communicating with care providers was preferred via personal contact and seemed to be the key element for user acceptance and for the successful implementation of a web platform like “ICareCoops” to support CBHC organizations.

## Introduction

Nowadays, older persons aged 65 years and older are still less familiar than younger generations with information and computer technology (ICT) usage. In Western societies, there is still a remarkable generation gap. In addition to age, other social and environmental determinants, such as reliable internet access, play a role in the use of ICT. Nevertheless, the percentage of ICT users among persons over 65 years has been rising in recent years [1-3]. Older persons are more likely to need support in daily life to stay at home independently because of morbidity, immobility, or general frailty. In the coming years, the proportion of the population aged 60 years and older will increase worldwide, specifically one in five people will be aged 60 years or older in 2050 [4]. Because of this demographic development, dignified and healthy aging and caregiving is becoming a more relevant topic in the long-term perspective as European health care systems are facing challenges of sustainability and an increasing need of resources [4,5]. As a consequence of these demographic changes, not only will the demand for health care services for older persons in need of support increase but a coordinated cooperation of services will be necessary to maintain seniors’ autonomous living at home [4,6]. In particular, ICT has a growing potential to provide essential assistance for older persons in need of support in finding and coordinating individual requirements for assistance with living and health care services. These ICT tools still have to be developed and implemented in a way that is consistently user-friendly yet adaptable for evolving health care models [6-8].

Besides public health care services, more and more nonprofit organizations are becoming major players in delivering health care services in communities [9-11]. Nonprofit organizations are based on five criteria, which are (1) organized, (2) private, (3) not profit-distributing, (4) self-governing, and (5) noncompulsory [10]. These mainly community-based organizations deliver a broad spectrum of social and health-related services, such as providing informal counselling and social support, building individual and community capacity, coordinating health services, and supporting self-management in older persons in need of support [12]. A well-known example of a nonprofit organizational form, which also comprises community-based health care (CBHC) services, are care cooperatives. The difference between cooperatives and other forms of enterprises is that profit-making and economic stability are balanced by the interests of the community [13]. Cooperatives are autonomous associations of people who voluntarily cooperate for their mutual social, economic, regional, and cultural benefit. Cooperatives balance multiple stakeholders’ interests instead of shareholders’ interests alone [9]. Cooperatives as a person-centered business model can become health care actors with the potential for large cost savings, service improvement, and more sustainable cooperation between those responsible for health care.

To date, care cooperatives in Europe have been established mainly in Italy, Turkey, France, and Spain [14]. In Switzerland, there are some care cooperatives (eg, Spitex, a Swiss nonprofit outpatient care cooperative [15]) and medical centers in rural areas. In the past, some of the cooperatives developed into good practice examples, whereas others failed because of poor communication, coordination, etc. Especially for these reasons, ICT tools could have a large impact on improving their services, addressing not only the concrete needs of older persons in need of support but also the daily operations of such organizations. Although the care cooperative model seems to be highly suitable for CBHC services, in this study we did not focus on one specific organizational form of CBHC service in the nonprofit sector. Instead, we focused on different CBHC organizations to identify the potential benefits of ICT support in their daily operations. Web platforms are a useful tool, but to the authors’ knowledge they have not yet been broadly implemented in CBHC organizations.

This study is part of a larger project, the European Active and Assisted Living (AAL) project called “ICareCoops”, that is targeted at CBHC organizations and aims to develop a web platform [16] for implementing and managing CBHC services for older persons in need of support. From the beginning, high user acceptance of the web platform has been set as the project’s focus. Therefore, a user-centered approach was chosen, which is characterized by heavy involvement of future web platform users. This user-centered design optimizes future ICT user participation, and it is a common method to develop digital health-related tools [17,18]. This study is one part of the web platform development.

The objective of this study was to explore concepts, approaches, and workflow processes of CBHC organizations and their potential as care cooperatives. Specifically, we pursued a two-part goal: (1) to achieve a comprehensive understanding of extant services offered and relevant requirements including risks and challenges of using ICT, and (2) to support these services with ICT solutions, exemplified in two European countries (Switzerland and Slovenia).

## Methods

### Study Design

To address the study’s goal, a user-centered design based on participatory research was chosen. Moreover, by using focus groups (FGs), a qualitative methodology was applied to identify the most relevant requirements from the users’ perspective. FGs were useful to identify the needs of target groups and to gather a broad spectrum of information and opinions from older persons in need of support. In our case, stakeholders comprised the following three groups: (1) older persons in need of support as care receivers of a CBHC service, (2) significant others of older persons in need of support, and (3) managers or care providers of a CBHC service in Switzerland or Slovenia. Discussing issues with multiple participants of the same stakeholder group reveals a broader variety of relevant aspects and useful ideas [19]. All participants were informed about the project and provided consent to participate in this study based on written and spoken information. FGs were treated with full confidentiality, and anonymity was maintained throughout the research process.

In qualitative studies, the role and reflexivity of the researchers is an influencing factor during the entire research process. Actions, perceptions, and especially context-relevant aspects are important and considered in the studied field—in this study, future users of web-based CBHC services for older persons in need of support. In qualitative research, the subjective perceptions of the researchers and users as a component of knowledge are applied to understand complex contexts [20].

**Figure 1:**
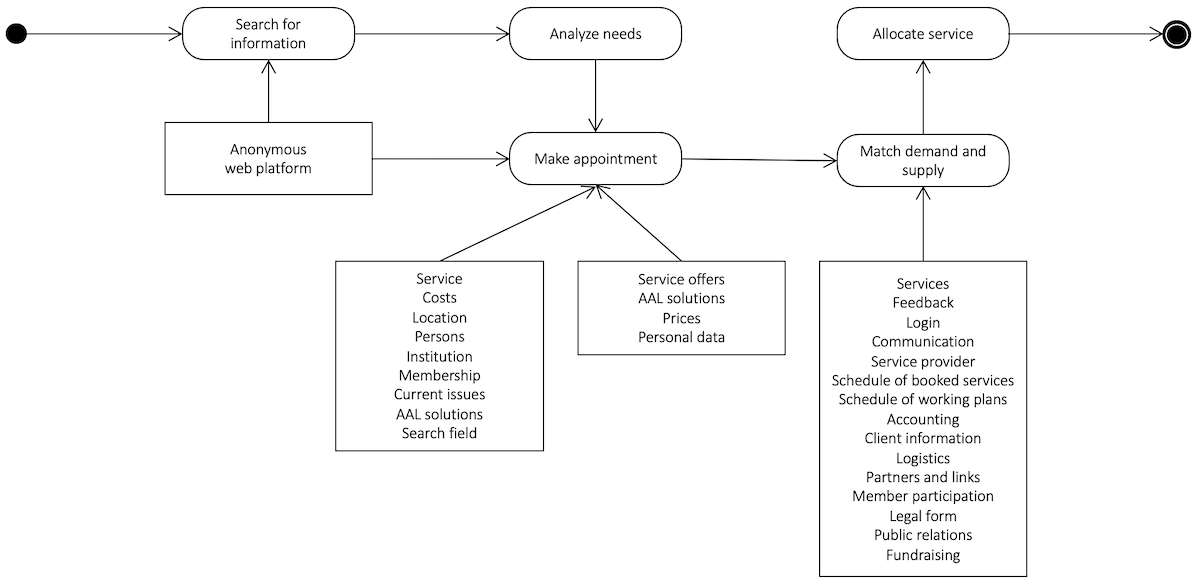
Workflow model of a community-based health care organization, including potential tools for support from a web platform. AAL: Active and Assisted Living.

To address all objectives within this qualitative study, we chose a mix of deductive and inductive approaches. The deductive component consisted of applying a workflow model (Figure 1) to demonstrate to the FG participants a potential template of a web platform and to discuss the different processes of a workflow within a CBHC organization in depth. Illustrating this simplified workflow model should enable tech-novice FG participants to discuss potential workflows that could be supported with ICT tools. To comply with the user-centered approach, the workflow model intentionally used everyday speech and its design was kept simple. In addition, the inductive component, with open-ended questions, facilitated gathering further information about FG participants. Detailed information on the data collection process is described further below under the “Data Collection” subsection.

### Sampling

For each FG, approximately 9 participants were invited by email and phone to join the FG to represent the needs and expectations of all three stakeholder groups. We considered differences in the requirements between the two countries, in the characteristics of the health care organizations (eg, year of foundation, size, ICT use), and between different stakeholders (older persons in need of support and their significant others and managers/care providers). To avoid the predominance of input from professionals, as well as mixing up knowledge and expectations of managers/care providers and older persons in need of support, the web platform requirements were discussed separately from three perspectives. Among the stakeholders, a heterogeneous group composition of participants was attained, allowing the collection of richer information [19].

### Recruitment Strategy

Participants from different economic and cultural backgrounds were consecutively selected in Slovenia and Switzerland. Swiss participants were recruited from the German-speaking region of Switzerland. In Slovenia, organizations located around the city of Ljubljana were contacted. In both countries, potential participating CBHC organizations received information about the project and were requested to distribute the invitation to their members. Furthermore, we asked them to send managers from an institution. We also ensured to include a variety of technologically competent people. The intention was to gain a better insight into the variety of workflows and requirements needed for the envisaged web platform.

### Ethical Approval

In Switzerland and Slovenia, ethical approval is required for clinical trials—for extraction of biological material or collection of health-related data [21,22]. This project is primarily concerned with the technical development of a software solution. End users were invited to contribute to the process of requirements engineering of software solutions. Given the nonmedical context, ethical approval was not required for all involved countries. Collected data were used exclusively for generating software requirements and improving the prototype. No risks or damages were foreseen during the end user FG study. Personal data of the end users was anonymized, codified, and stored in a secure place, guaranteeing access only to authorized persons and safeguarding the right to privacy. The software prototype does not contain any personal information from end users. Informed consent highlighted the possibility of research results being published in scientific journals or being presented at conferences, always with the guarantee of anonymity. All participants had an exit right and could withdraw from the project at any time without giving a reason.

### Data Collection

To gather sociodemographic data from our sample, participants were asked to complete a questionnaire. During the FG process, we followed a semistructured topic guide with four open-ended questions. All four questions were based on scientific literature searches, including best-practice models of workflows and web platforms of CBHC organizations.

For this study, we developed a simplified workflow model as a basis for discussion with the stakeholders (Figure 1). This model comprised the most relevant four steps within the workflow of a CBHC organization and expected stakeholder requirements. Subsequently, we tried to combine the consecutive workflows within the potential web platform of a CBHC organization:

1. Need for care support: from a user perspective, the workflow starts with an internet search.
2. Information: relevant information has to be presented on the web platform concerning the organization (eg, name, membership, region, services, etc).
3. Needs analysis: the workflow continues by contacting the CBHC organization and making an appointment to assess the demands of the user. In this step, we wanted to know which kinds of ICT users would use (eg, email, telephone, video call, apps, etc).
4. Allocation of services: after an assessment of needs, services had to be selected and offered.

The model considered the three different stakeholder groups. For all three target groups, needs for working and cooperating in a CBHC organization with a variety of requirements (eg, price checking, membership management, scheduling, communicating with others, etc) were discussed.

This workflow model was one component of the FG topic guide. Furthermore, the guide included an introductory section about the project, study-specific open-ended questions pertaining to users’ needs and expectations, time for further issues, and an outlook of the following project steps.

Study-specific open-ended questions used to derive user-specific requirements, expectations, and challenges related to ICT-based support of a CBHC organization are illustrated in Table 1.

**Table 1:** Open-ended questions in the topic guide for focus groups from three perspectives.^a^

Topic	Questions
Identify workflows including ICT^b^ solutions	Which other services would be useful in your organization? If you think long term, which services could be relevant in the future?
Identify desirable ICT solutions	Which ICT supports do you think would be helpful to care organizations? Which functions should be possible?
Identify barriers to ICT usage	Which barriers/risks do you identify in the usage of ICT solutions? (eg, cultural, ethical, legal—personal and general)
Identify potential of a care cooperative	Name the 2 to 3 most relevant advantages of founding a care cooperative from your point of view.

^a^Perspectives of older persons in need of support as care receiver, significant others of care receivers, and managers or care providers of community-based health care organizations.

^b^ICT: information and computer technology.

Four researchers conducted the FGs—two in Switzerland and two in Slovenia. One researcher acted as moderator and one assisted by taking field notes. To guide the discussions and focus on the topic, questions were presented as PowerPoint slides. A duration of 2 hours for one session was not exceeded. Each FG session was audio-recorded and transcribed verbatim after receiving written consent from the participants. In all transcripts, protocols, and case reports, names and personal data were anonymized.

### Data Analysis

**Figure 2:**
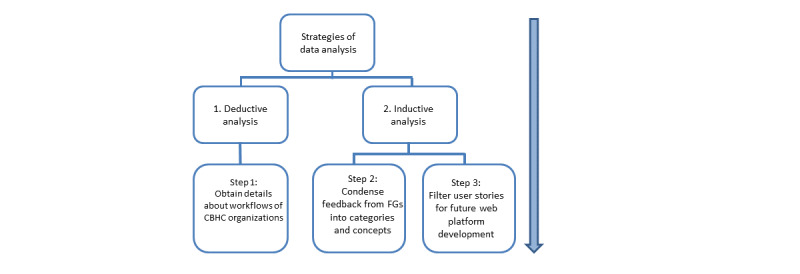
Strategies and steps of focus group data analysis. CBHC: community-based health care; FG: focus group.

Data were extracted by thematic content analysis based on written transcripts, which was conducted in three steps, characterized by an inductive and deductive strategy to receive two different kinds of data (Figure 2). In step one, the deductive data analysis, we aimed to obtain details about the workflows of CBHC organizations. Statements and responses from FG participants were summarized according to the topics in the workflow model. In step two, the inductive data analysis, information, new themes, and issues mentioned by FG participants were first read in-depth, open codes were generated, and the data were condensed into concepts and categories. Microsoft Excel 2010 was used for the structured data analysis. In step three, user platform requirements had to be filtered systematically for further web platform development. Based on the results of steps 1 and 2, technically relevant results were translated into user stories of the syntax “<as an> <I want to> <so that>.” The user stories will be the basis for further technical web platform development in the future and were not part of this study, which focuses on an analysis of specific needs of potential web platform users. User stories are a method that was applied to gather user requirements for developing an agile web platform [23].

### Quality Assurance and Trustworthiness

Data security regulations regarding data storage were observed throughout the study. For data quality assurance, the Consolidated Criteria for Reporting Qualitative Research (COREQ) checklist was applied [24]. For peer review, a pretest of the FG questions in the topic guide was conducted. Field notes were collected by a second researcher. After each FG session, both researchers reflected on the process for potential improvements with a written debriefing. For peer review, a second researcher coded 25% of the data from the transcripts. The results were discussed in order to reach consensus on each concept. All participants of the study received feedback in the form of a plain-language summary of the study results via email.

## Results

### FG Sessions

In Switzerland and Slovenia, six FG sessions, each with a maximal duration of 2 hours, were conducted with three target groups: (1) older persons in need of support as care receivers of a CBHC service, (2) significant others of older persons in need of a CBHC service, and (3) managers or care providers of a CBHC service. Of the 10 organizations who were invited, 70% (n=7) participated in the FGs by sending different stakeholders: managers, care providers, older persons in need of support, and significant others of older persons in need of support.

### Sociodemographic Data

In total, six FGs with 40 participants (32 women, 8 men) were conducted. Personal characteristics indicated a large variety of tech-savvy and non–tech-savvy people and formal and informal care providers and receivers and their significant others. Sociodemographic data of FG participants are presented in Table 2.

**Table 2:** Sociodemographic data from the three target groups in the focus groups.

Country of data collection and target group		Participants, n	Females, n (%)	Age (years), median (range)	Characteristics of sample
**Switzerland**					
	Older persons in need of support	7	5 (71)	75 (67-83)	Tech-savvy and non–tech-savvy; formal and informal care receivers; potential care receivers; members of CBHC organizations and non-members
	Significant others of older persons in need of support	7	4 (57)	64 (53-68)	Tech-savvy; informal care providers and potential informal care providers for family members
	Manager/care provider	8	5 (63)	71 (65-80)	Tech-savvy and non–tech-savvy; informal care providers; management members of CBHC organizations
	Total	22	14 (64)	70 (53-83)	
**Slovenia**					
	Older persons in need of support	4	4 (100)	72 (69-83)	Non–tech-savvy; basic education; low-income; formal care receivers
	Significant others of older persons in need of support	7	7 (100)	37 (28-58)	Tech-savvy; informal care providers and potential informal care providers for family members
	Manager/care provider	7	7 (100)	65 (59-79)	Tech-savvy and non–tech-savvy; informal care providers; management members of CBHC organizations
	Total	18	18 (100)	64 (28-83)	
**Switzerland and Slovenia (total)**		40	32 (80)	67 (28-83)	

### Deductive Analysis of Organizations’ Workflows

Participants from the manager/care provider group mainly agreed on the workflow model (Figure 1). Usually, older persons in need of support call the organization by phone and ask for services. In most cases, service providers initiate a personal appointment with the service receiver to identify needs to establish a personal, face-to-face relationship with the older person in need of support.

To our understanding the first contact has to be free of charge.

Relative of care receiver 03_Slo

Personal contact is central to all of our members. This will remain the most important rationale of CBHC organization beyond the next ten years.

Care provider 04_Swiss

After determining the patient’s needs, the organization arranges tailored services for the older person in need of support. The FG participants recommended involving older persons in need of support and their significant others in the decision-making process for care services. A test period of services would be beneficial for older persons in need of support. After this period, they could decide whether to continue with the services or not. Participants recommended that a contract should be signed between older persons in need of support and CBHC organizations to clarify arrangements, frequency of services, and payment terms. In most organizations, a person in the role of a distributor works in the office. Service users wished to have a contact person in the organization who could coordinate the requests and service offers. In a CBHC organization, a distributor is required to monitor services, contact older persons in need of support, and coordinate formal and informal care providers. Moreover, the same person determines conditions and deadlines of payments according to user needs and facilitates communication among all stakeholders. A participant in the provider FG in Switzerland emphasized, “The organization would not work without a contact person who coordinates all the stakeholders’ needs” [Care provider 02_Swiss].

### Inductive Analysis

In the inductive analysis, five main categories were extracted from open coding of data from the three stakeholder groups: (1) ICT usage behavior of users, (2) challenges of web platform usage, (3) content and technical requirements for the web platform, (4) form and services of CBHC organizations, and (5) rationales of CBHC organizations. Each category is described in the following sections, including examples of codes. The results of categories 1, 4, and 5 are illustrated in Table 3. The results of categories 2 and 3 are presented separately in Table 4 and Multimedia Appendix 1, respectively.

**Table 3:** Subcategories and codes of three of the five main categories extracted from the study data.

Category and subcategories		Codes
**ICT^a^ usage behavior**		
	Current ICT usage among older persons	ICT usage rises in the next 10-20 years No idea about options of ICT Significant others more familiar with ICT usage Small proportion of the elderly is familiar with ICT Open-minded attitude regarding ICT usage No information accessible without the internet
	Current ICT usage among CBHC^b^ organizations	Use of ICT via phones, Excel, and voicemails Computer cannot substitute a person or individualize the services Coordination of services is performed by people (ie, not possible without them) ICT usage by managers of organizations, only by a few members
	Usage of web platform	Benefit greater for managers of CBHC organizations Participants would be interested in using the web platform Future generations to benefit more
**Form and services of CBHC organizations**		
	Organizational form: association or cooperative	Associations as business models are more represented than cooperatives Liability problems are excluded in associations Associations allow spontaneous freedom of action No difference between associations and cooperatives
	Current and desirable services of CBHC organizations	Independent counselling without the intention of selling something Carpools General practitioners and health professionals in care cooperatives Deposit of private data, such as patient’s provision or testaments Individual services adjusted to available budget Driverless vehicles (AAL^c^ project) Rental robots (AAL project) High-quality meals-on-wheels Support in emergency situations (eg, correct phone numbers, weekend assistance) Coordination of professional care Expansion and optimization of palliative care
	Possible concepts for the work of CBHC organizations	Simple neighborhood aid Mixed generations (mutual aid) Living concepts for mixed generations Older persons as informal caregivers (mutual aid) Refugees as informal caregivers
**Rationales of CBHC organizations**		
	Rationales together with older persons in need of support	Inhibition in asking for support (neighbors, family) Concerns regarding nursing homes Physical and mental limitations normal Demand required for internet usage Nonacceptance of support Societal changes, disruption of family structures Personal contact very important (prevent social isolation) Autonomy as long as possible
	Rationales collaborating with care receivers/members of the organization	Never say no to member requests Social contact is necessary for needs assessment Need to talk to someone, as social contacts decrease Personal conversations essential Care requires consideration of logistics (coordination is important) Social contacts arise spontaneously
	Rationales cooperating with volunteers	No bureaucratic effort, less scheduling Motivated volunteers are available No exploitation of volunteers Recompense for volunteers (eg, time credit, tax reduction)

^a^ICT: information and computer technology.

^b^CBHC: community-based health care.

^c^AAL: Active and Assisted Living.

**Table 4:** Subcategories and codes of main category 2: challenges of web platform usage.

Subcategories	Codes
Common issues	Information overload on the internet (or on the web platform, respectively) Transmission time of the internet can be slow The knowledge gap in using the internet has to be filled in the target group Technical products (AAL^a^ project) and requirements change very quickly nowadays
Ethical issues	Protection of sensitive data has to be respected User awareness that the internet works in the same way as a monitoring system has to be raised Concerns about moneymaking with the web platform Health concerns have to be respected (people with dementia cannot use the platform and book services)
Financial issues	Not everyone can afford internet access Not everyone can afford the required hardware (smartphone, PC, tablet, AAL) Retired people cannot afford any additional expenditures (services, hardware) There has to be a wide range of payment options (credit card, PayPal, bank transfer) Risk for seniors of buying/ordering too much without control
Cultural issues	Not everyone has access to the internet (especially this target group) Some people refuse to use the internet/TV, etc Target group is afraid of the future regarding internet usage (and its possibilities) Owning a smartphone is more common than owning a computer (ie, apps would be more helpful than PCs)

^a^AAL: Active and Assisted Living.

#### ICT Usage Behavior of Users

In this category, the target groups’ current ICT usage behavior was compiled. All statements concerning the issue of ICT usage of seniors supplement this category.

Researchers recognized a great variance in tech-savviness among participants. The user (older persons in need of support as care receivers) FG in Switzerland was more tech-savvy than managers or care providers. In contrast, the user group in Slovenia did not have any experience in using ICT. Thus, all FGs were conducted on a very different level of understanding regarding the development of a web platform. Overall, participants assumed that modern ICT cannot be used by approximately 50% to 80% of older persons in need of support because of lacking skills or familiarity with ICT usage. At present, only a small sample of tech-savvy seniors would benefit from such a web platform, although future generations may benefit from the web platform. A period of about 10 to 20 years is predicted for older persons in need of support to become sufficiently tech-savvy to use a web platform.

Older persons in need of support would be interested in improving their skills and therefore suggested courses as a requirement for establishing this web platform. They also assumed that care providers would benefit more from such a web platform from a management and organizational point of view.

The most common reported uses of ICT were phone calls for making appointments, emails for confirmation of appointment dates, and Excel spreadsheets to collect information about care receivers/members/providers. CBHC organizations knew that current ICT usage is very poor, but they did not have resources to invest in this issue and were satisfied with their current state. Most of the FG participants had difficulties in anticipating future needs and providing innovative ICT solutions; however, they all demonstrated an openness to using such a web platform if it was user-friendly and easy to access and navigate.

#### Challenges of Web Platform Usage

Participants were asked to discuss challenges of using the web platform. The identified barriers could be divided into four subcategories: (1) common issues, (2) ethical issues, (3) financial issues, and (4) cultural issues (Table 4). Low ICT usage among the target group of seniors was seen as a main challenge for the web platform; they would assume there to be an information overload on the internet and would therefore only use an easy and clearly arranged web platform.

If I go online and search, for example, meals-on-wheels, I will find so much information and I can’t handle this information. I need only some high-quality results in the region where I live. If the web platform could filter this information for my needs and was easy to use and clearly structured, I would appreciate this web platform.

Care receiver 04_Swiss

Furthermore, trust in data protection among the target group was critical. They reported being afraid of storing sensitive data on a web platform.

#### Content and Technical Requirements for the Web Platform

As a main result of FG discussions, specific requirements for the web platform were mentioned and could be retrieved. The requirements covered common, technical, and content aspects of the web platform in regard to hardware and mobile apps; these requirements are summarized in Multimedia Appendix 1.

The ICT system should be very well built, and someone should monitor it and organize the work. For the care-providing organization, it is important to have good visibility and access to data.

Care provider 03_Slovenia

All of the participants confirmed that there need to be people in the background to organize and manage all of the services and member requests; one participant in Switzerland called it an “intelligent system” [Relative of care receiver 01_Swiss]. All participants were quite open-minded regarding hardware and potential software solutions offered. They mentioned possibilities such as fingerprint identification, voice-guided tools, video calls, and video tutorials to explain the web platform. In total, participants named 60 requirements, which were then translated into user stories for further web platform development in a separate step of the project. The separate step is not part of this study. Examples of user stories include the following:

I as a care provider have the option to contact the care receiver via an app, so that it’s possible to inform about changes in the schedule, etc.

I as a care receiver have video tutorials which explain important information (service), so that I understand and get an idea of the services offered or other important information (how can I become a member, how can I offer help…).

Overall, the idea of CBHC organizations working with such a web platform was very well received, with the prerequisite that it would be user-friendly and accessible without a high cost.

#### Form and Services of CBHC Organizations

This category includes information concerning the business model of a CBHC organization comparing associations and cooperatives. The main task of a CBHC organization as a service provider is to fulfil the target group’s needs, which are also described by this category. For care providers, the organizational model of cooperatives is currently not widespread in Switzerland. A widely used business model for existing institutions is an association because it is easier to administer (eg, regarding liability of partners). Differences between acting as an association and acting as a cooperative in health care were not obvious to FG participants.

It is easier to work as an association in Switzerland than as a cooperative as legal form for various reasons.

Care provider 01_Swiss

Nevertheless, the motivation to work with cooperative principles is untouched by this solution. Slovenian participants emphasized that they did not have cooperatives in Slovenia, but they assumed it was an engaging idea. In general, the suggestion of care cooperatives was convincing to participants in both countries.

The variety of current and desirable service offers in CBHC organizations is very broad and ranges from collective buying (eg, of medications) to assistance in housekeeping. Organizations offer an endless number of different services, except professional care. They do not offer health or nursing care because this is provided by special health care organizations. Participants mentioned the desire to simplify health care by including professional care and practitioners to coordinate all care services offered by CBHC organizations. To give an impression of services offered, care organizations brought information materials to the group sessions; their services included mowing the lawn, accompanying someone to the doctor, transportation services, and special training for seniors, such as computer courses or universities for seniors. It was also identified that seniors can rarely afford additional expenditures, such as care services.

Therefore, the services have to be affordable even to people with low incomes; usually they are co-financed by municipalities.

Care provider 01_Slovenia

Moreover, dissatisfaction with professional care in health care organizations was revealed. Primarily for potential users of CBHC organizations, some services should be strengthened, such as high-quality meals-on-wheels, emergency aid at night and on weekends, and regional information for seniors convened at one place. In general, participants seemed to have a need for information about any issues related to seniors located in one place (eg, care, courses, communication, leisure, etc).

The CBHC organization could act as an information center.

Relatives of care receiver 03_Swiss

CBHC organizations should (1) inform users via different channels (internet, telephone, in person, printed documents) about any issues related to seniors; (2) provide customized recommendations for care needs; and (3) store data about users and transfer them to relevant stakeholders, such as care providers or practitioners. Furthermore, ideas about AAL solutions were suggested, such as robots to support housekeeping or fully automated, driverless vehicles.

Participants discussed possible concepts for CBHC organizations to involve retired people in the informal care of seniors as well as intergenerational projects. Younger generations could assist older persons in need of support, and older persons in need of support could assist families in other areas of need (eg, babysitting). Furthermore, living concepts, where seniors live with younger generations in the same housing facility, were mentioned as desirable.

#### Rationales of CBHC Organizations

This category summarizes statements of the target groups about fundamental principles to be considered in CBHC organizations, as they seem to be of particular importance for collaboration with older persons in need of support.

Participants mentioned rationales for the collaboration of CBHC organizations with older persons in need of support; participants reported that autonomy was desirable if possible. Therefore, a decrease of physical and mental mobility is seen as an obstacle, as it could potentially lead to a loss of autonomy. Nowadays, family structures are supposed to be less stable and close so that older persons in need of support do not require support from the family.

I don’t want to disturb my children. They are very busy with a lot of other things.

Care receiver 06_Swiss

Beyond that, older persons in need of support worry about moving to nursing homes. Here one can see a discrepancy between the intention to live independently, the fear of requesting support or moving to a nursing home, and the need for support caused by a decrease in physical and mental mobility. Participants confirmed that they needed support when using the internet; they would probably need supervision when using ICT.

Nearly all FG participants ranked having a personal contact within a CBHC organization as the most important feature. Older persons in need of support need to have a contact person within the organization that they refer to. They assumed social isolation would be a risk factor as ICT use increases.

It is not an exception that service providers and mediators of the informal care organizations are the only contact persons that seniors still have.

Care provider 01_Swiss

Booking “social contact” as a service offering is perceived as taboo. For many, social contacts are to be established spontaneously and not by booking an appointment. Participants gave a clear definition of when personal contact to organizations is required: as soon as the care service becomes intimate (ie, someone coming into their house or taking over intimate care services like hygiene).

A further principle of all participating care organizations reported by participants is

… to provide as much support as possible to assist members. Usually we don’t say NO to our members.

Care provider 08_Swiss

CBHC organizations tend to decline none of the requests of their members. In their daily business, they feel challenged and responsible for creating new solutions, also for nontypical and nonstandard needs.

Participants confirmed that there are volunteers motivated to act as informal caregivers, but efficient ways of collaboration need to be established. This could be achieved by paying overtime or monetary credits for volunteers. Collaboration should work with small bureaucratic effort for volunteers. Nonfeasible expenditure/investment would exclude this important group of informal care givers.

## Discussion

### Principal Findings

This qualitative FG study aimed to explore concepts, approaches, and workflows of CBHC organizations in order to achieve a comprehensive understanding of existing services offered and relevant requirements to support these services with ICT solutions exemplified in two European countries. The Swiss and Slovenian results are related to three stakeholder groups: (1) older persons in need of support as care receivers of a CBHC service, (2) significant others of older persons in need of support, and (3) managers or care providers of a CBHC service. Results from the deductive analysis of existing workflows of CBHC organizations and the potential need of ICT support in the different workflows will be discussed, as well as the results from the inductive analysis based on five main categories: (1) ICT usage behavior of users, (2) challenges of web platform usage, (3) content and technical requirements for the web platform, (4) form and services of CBHC organizations, and (5) rationales of CBHC organizations.

To conclude, the idea of CBHC organizations delivering formal and informal care is very popular and is recommended among stakeholders. Participants emphasized personal contact between members and CBHC organizations as a main rationale for CBHC organizations. Social contact is seen as highly important to older persons in need of support. The main challenge when using a web platform such as the one being proposed will be the knowledge gap in ICT usage among older persons in need of support. Therefore, an age-appropriate web platform design and seniors’ mistrust in data protection need to be addressed. ICT usage will likely rise during the COVID-19 pandemic, but it can also lead to even larger “digital divides” among older persons in need of support.

Results of technical and content-based requirements for the web platform showed the openness of potential users regarding hardware and software solutions. FG participants emphasized the importance of an “intelligent system” behind the web platform in the form of a person or organization that coordinates and leads the web platform. The workflow of a CBHC organization proposed by the project group (Figure 1) was largely acknowledged by FG participants. Furthermore, it can be concluded that stakeholder results between different FGs were widely congruent. Certainly, there were differences in the aspects discussed, as there were three different stakeholder perspectives, but they shared the same ideas about principles of CBHC organizations, challenges of ICT usage, and the content and technical requirements for the web platform. Major differences in the issues mentioned by the different FGs were noted in the results section and will be discussed further below.

### ICT Usage and its Challenges

A main issue in the discussions about the web platform was the lack of knowledge of ICTs and the habits and skills of older persons in need of support with ICT applications. Apparently, older persons in need of support as care receivers and managers or care providers of CBHC organizations could not imagine the possibilities of ICT. One of the reasons why a third stakeholder perspective—of the significant others of care receivers—was needed in this study was because the younger generation is expected to be more tech-savvy and will profit more from such a web platform. In Switzerland, older persons in need of support as care receivers confirmed that many of them were quite familiar with using ICT daily for information seeking and communication with family and friends. It was easier for them to imagine a web platform that contains tools to support CBHC organizations. A reason for high tech-savviness of Swiss older persons in need of support could be that those interested in using ICT may follow an invitation to a discussion on this topic rather than care receivers who are unfamiliar with ICT. In Slovenia, in contrast, the older persons in need of support were not familiar with ICT usage. This helps to gain a broad understanding of the variability of ICT usage among this target group in Europe. Daily internet usage in Switzerland is slightly above the European Union average [1].

Managers and care providers insisted on their current concepts and workflows in their CBHC organizations and could not imagine a web platform supporting and coordinating members and care providers. Older persons in need of support were aware of their knowledge gap and lack of practice in using ICT. They were convinced that future generations would benefit from this web platform. Moreover, they were open-minded and interested in taking ICT courses to improve their skills. These results are confirmed by the literature. In Switzerland, 41% of people aged 70 years and older use the internet, whereas 97% of people younger than 30 years of age use it [1]. Internet use on a frequent basis (several times a week) is common for 34% of people older than 65 years [2]; the rate in this age group has more than doubled within the last year and is increasing [2,25]. The United States is often seen as a pioneer regarding modern technologies. Already one-half of its population over 65 years uses the internet [3], which can be seen as a prediction for Europe. According to the Nielsen Norman Group [26], approximately 65% of people in the United Kingdom aged 65 to 74 years are using the internet. Older persons in need of support in Europe use the internet primarily for seeking information. Bilateral communication and social interaction via blogs, social networks, and web communities are not yet common among older persons in need of support living in Europe. Only about 10% of older persons in need of support use web 2.0 tools [27]. However, in the United States, 34% of people older than 65 years use social networks for communicating with family and friends [3]. Therefore, it is not surprising that participants in this study are not yet familiar with the potential of ICT. According to the literature, web accessibility currently faces three key challenges: (1) the web is growing faster than accessibility efforts progress; (2) as content, presentation, and design of websites are getting more sophisticated, so must technical skills; and (3) the rise of user-friendly (social) web platforms enabled non–tech-savvy people to share huge amounts of data online and most of them are in inaccessible formats [28]. These aspects should be considered when developing a web platform for older persons in need of support.

Hence, FG participants revealed many challenges when using this web platform. The main obstacles were ethical issues, such as mistrust in data protection and privacy concerns; financial issues, such as costs to purchase hardware and for internet connection; cultural issues, such as age and the knowledge gap for ICT usage; and common issues, such as the assumed information overload on the internet. These findings can be confirmed by the literature [29-31]. The study revealed frustration by users, which can result from searching for information—for example, when a Google search yields too many results. Furthermore, mistrust in the search results was a problem for users. They did not know how to select relevant information. This means that resources and knowledge of quality standards are currently lacking. These challenges of older persons in need of support regarding internet searching are congruent with the literature [8].

One additional factor was named in this study: participants identified social isolation to be a risk for older persons in need of support, which would rise with increased ICT usage. This aspect was mentioned in the context of cooperatives and challenges of using the web platform or ICT together. Furthermore, participants were afraid of older persons in need of support using apps to book a service or to talk to care providers via Skype instead of through a personal, face-to-face conversation. They confirmed that CBHC organizations or care providers in some cases are the last remaining contact that care receivers or older persons in need of support have. Therefore, they ranked personal contact in a CBHC organization as the most important component. They reported that booking personal contact as a service feature was considered taboo. In the literature, there is no agreement on whether social isolation and loneliness increases or decreases as a result of internet use [32]. Various research results indicate that internet usage is connected to a reduction of social isolation among older persons in need of support and increased well-being [33-36]. A decrease in loneliness and increase in social contacts were recently confirmed as benefits of internet usage for people living in assisted living communities [32]. These studies were mainly conducted in the United States. The low rate of internet usage behavior among older persons in need of support in Europe could result in a lack of unawareness of the possibilities related to ICT usage. Intensifying training classes for older persons in need of support can promote ICT usage, especially communication via web 2.0 tools. This could lead to a decrease in social isolation among older persons in need of support. It was especially important for the project team to obtain input from potential web platform users. We were reassured that CBHC services focus on individual contact. The anxiety of older persons in need of support that social isolation may rise with internet usage has to be considered. Recent research shows opposite outcomes, which reveal improved well-being and a decrease in social isolation [32]. Therefore, fostering an increase in ICT usage in older persons in need of support and supporting their growing ICT skills needs be an aim of today’s society.

### Challenges for CBHC Organizations

In addition to issues related to ICT, stakeholders also identified more general aspects regarding challenges for CBHC organizations. Organizations involved in the study offer a wide range of services, from accompanying someone to the doctor to mowing their lawn or cleaning their house. One of their principles seems to be to find solutions for any requests from their members or older persons in need of support. In both countries, Switzerland and Slovenia, only informal care was delivered by CBHC organizations. The reason might be the existing structures in the health care system, where professional care is delivered by special outpatient nursing services, such as Spitex in Switzerland, which is paid by insurance companies, the state, and care receivers [15]. The stakeholder group of significant others of care receivers emphasized that one inclusive organization, which offers and coordinates care services of care receivers, would be preferable.

One further issue is the legal form of CBHC organizations. Participants of this Swiss and Slovenian study were predominantly CBHC organizations acting as associations because only a few genuine care “cooperatives” exist in these two countries [37,38]. The International Cooperative Alliance defines a cooperative as an “autonomous association of persons united voluntarily to meet their common economic, social, and cultural needs and aspirations through a jointly-owned and democratically controlled enterprise” [39]. Cooperatives as an organizational form are less recognized in the health care sector in Europe, despite some examples from Italy [40-42]. In Switzerland and Slovenia, cooperatives in the health care sector are still uncommon. Instead, associations act like cooperatives. In Switzerland, for instance, an adaptation of the law would be necessary to enable organizations to found cooperatives and facilitate their work [10]. In Slovenia, the cooperative law was adapted several times, most recently in 2009, when the European cooperative legal order was introduced to Slovenian law [11]. This could be a potential explanation for the small number of existing care cooperatives in Slovenia.

### Limitations

While many helpful requirements and needs were identified to develop the platform, some limiting aspects of the study have to be considered. One aspect concerns the study design. The overall framework of the project restricted the number of FGs and involved countries. Nevertheless, the heterogeneity of the countries was satisfying because Switzerland is, in contrast to Slovenia, one of the leading countries concerning technology in Europe. The majority of FG participants being women reflects the societal phenomenon that women are still more involved in caregiving, professionally and informally [43]. Therefore, the FG sampling is assumed to be comparable with the status quo of CBHC organizations. Based on these framing project conditions, the criteria of saturation could not be applied for data collection. Thus, the question cannot be answered whether additional FGs from the same target groups and countries would have yielded more or different information. In the end, one could assert that the FGs revealed enough information because we did not notice any additional information gain from Slovenia compared with Switzerland.

Participants mainly agreed on the proposed workflow model, which was the basis for the FG discussions. Nevertheless, this abstract workflow was a complex model that was not easy to understand for the participants. To compile the lessons learned, more time is required to develop an in-depth understanding and discussion of outcomes. Therefore, we focused the discussions on the questions of the topic guide.

### Conclusions

The study revealed a complex variety of results; some of the major issues are summarized below:

- Workflows of CBHC organizations need to be simple and congruent with the discussed workflow model (Figure 1):
  older persons in need of support and their significant others need information presented in a user-friendly way about the CBHC organization and their services;
  at best, relevant information about the needs of older persons in need of support from a specific region needs to be presented on the CBHC web platform; and
  older persons in need of support and care providers need applications to establish contact with each other. Potential tools can vary from telephone calls and personal meetings to online video calls or chat applications.
- Although an overall openness regarding ICT was identified, ICT usage behavior varied enormously among FG participants. This makes it even harder to develop customized web platforms for this target group.
- Challenges of the potential web platform seem to be related to general concerns, such as data protection, access to the internet and ICT, and knowledge gaps in using ICT in the target group.
- The organizational form of CBHC organizations as associations is preferable because of low-threshold structure and establishment. The form of cooperatives is not common in the countries involved in this study.
- Rationales of CBHC organizations are quite clear: maintain a user focus, respect autonomy of older persons in need of support, and maintain personal contact between CBHC organizations and older persons in need of support.

In summary, we conclude that the majority of current stakeholders (older persons in need of support, significant others, and managers/care providers) are not familiar or experienced with ICT usage. Coming generations will contribute more concrete ideas of useful requirements and will benefit more from the web platform. For this reason, training for using the web platform and for setting it up for the specific CBHC organization will be offered. Older persons in need of support seemed very open-minded regarding training for ICT usage; therefore, existing offers of training should be intensified to meet the demands of the older persons in need of support. Once the target users are trained to use ICT, they can experience benefits from the web platform, such as decreased social isolation, independent living, and adequate user-oriented health care services. Further research is required in this context.

The web platform has the potential to facilitate the foundation, work, and collaboration of CBHC organizations in Europe. Overall, personal contact is a main request of older persons in need of support and managers and care providers; in Slovenia, it was cited by almost all participants. Searching for care services, contacting care providers, and communicating with care providers is preferred via personal contact and seems to be the key element for user acceptance and for the successful implementation of a web platform like “ICareCoops” [16] to support CBHC organizations.

## Appendix

Multimedia Appendix 1 Additional file requirements matrix.

## Abbreviations

AAL: Active and Assisted Living
CBHC: community-based health care
COREQ: Consolidated Criteria for Reporting Qualitative Research
FG: focus group
ICT: information and computer technology
